# Quantitative assessment of background parenchymal enhancement in breast magnetic resonance images predicts the risk of breast cancer

**DOI:** 10.18632/oncotarget.13538

**Published:** 2016-11-24

**Authors:** Xiaoxin Hu, Luan Jiang, Qiang Li, Yajia Gu

**Affiliations:** ^1^ Department of Radiology, Fudan University Shanghai Cancer Center, Shanghai, China; ^2^ Department of Oncology, Fudan University Shanghai Medical College, Shanghai, China; ^3^ Center for Advanced Medical Imaging Technology, Shanghai Advanced Research Institute, Chinese Academy of Sciences, Shanghai, China

**Keywords:** BPE, breast, quantitative assessment, breast parenchymal enhancement rate, MRI

## Abstract

The objective of this study was to evaluate the association betweenthe quantitative assessment of background parenchymal enhancement rate (BPER) and breast cancer. From 14,033 consecutive patients who underwent breast MRI in our center, we randomly selected 101 normal controls. Then, we selected 101 women with benign breast lesions and 101 women with breast cancer who were matched for age and menstruation status. We evaluated BPER at early (2 minutes), medium (4 minutes) and late (6 minutes) enhanced time phases of breast MRI for quantitative assessment. Odds ratios (ORs) for risk of breast cancer were calculated using the receiver operating curve. The BPER increased in a time-dependent manner after enhancement in both premenopausal and postmenopausal women. Premenopausal women had higher BPER than postmenopausal women at early, medium and late enhanced phases. In the normal population, the OR for probability of breast cancer for premenopausal women with high BPER was 4.1 (95% CI: 1.7–9.7) and 4.6 (95% CI: 1.7–12.0) for postmenopausal women. The OR of breast cancer morbidity in premenopausal women with high BPER was 2.6 (95% CI: 1.1–6.4) and 2.8 (95% CI: 1.2–6.1) for postmenopausal women. The BPER was found to be a predictive factor of breast cancer morbidity. Different time phases should be used to assess BPER in premenopausal and postmenopausal women.

## INTRODUCTION

Breast cancer is the most common female malignancy worldwide [1–3]. Effective early detection by imaging studies remains critical to decrease mortality rates, particularly in women at high risk for developing breast cancer. In the late 1980s, Gail developed a series of evaluations and a prediction model for high-risk breast cancer [4], which has been widely used. However, given the diversities of heterogeneity and carcinogenesis, simply assessing life style and family history using questionnaires was far from enough to predict morbidity [5].

Mammographic density has been related with the risk of developing breast cancer, and this parameter has been widely used for the prevention and early detection of breast cancer [6, 7]. The risk of breast cancer in women with high mammographically breast dense is about 3 to 5 times higher than that with predominantly low mammographically breast dense. However, further studies indicated that three-dimensional (3D) tomography would be more accurate in evaluating breast density than the two-dimensional data obtained by mammography [8].

King et al. and Dontchos et al. examined the relationship between breast cancer and both the amount of fibroglandular tissue (FGT) and level of background parenchymal enhancement (BPE) using magnetic resonance imaging (MRI) [9, 10]. Their results suggested that greater BPE was associated with a higher probability of developing breast cancer. The odds ratio (OR) for moderate or marked BPE versus minimal or mild BPE was 10.1. Moreover, BPE findings remained significant after adjustment for FGT. This may be because BPE is partly an evaluation of the blood supply to breast tissue. They did mention, however, that there was observation bias in the subjective evaluation of BPE by physicians. Additionally, only early stage BPE was measured, and the middle and late stage BPEs were not evaluated in their studies. Kajihara et al. analyzed the relationship between enhancement time phase and BPE. They found that BPE was significantly stronger at the delayed phase than at the early phase in both qualitative and quantitative assessments throughout the menstrual cycle. [11] Thus, for better assessment of BPE in multiethnic populations and different centers, quantitative BPE should be used for accurate, objective and reproducible results.

Added to the time phase-dependent diversity, BPE is affected by hormones, age, menopausal status, menstrual cycle, the use of tamoxifen or aromatase inhibitors and hormone replacement therapy [11–16]. Thus, we developed software to assess BPE automatically. In this study, we aimed to evaluate the positive predictive capability of the quantitative assessment of BPER in breast magnetic resonance imaging (MRI) to determine the risk of developing breast cancer among women with breast cancer and benign lesions, as well as controls, with our newly developed software.

## RESULTS

### Characteristics of the study groups

There were 101 patients each in the breast cancer, benign lesions and control groups (Table 1). The mean age in each group was similar according to the paired-matching principle: 49.2 (24–78) years, 48.6 (24–78) years, and 48.8 (26–74) years. There were 47 premenopausal patients in each group, among which 8 (16%) were in the first week of menstrual cycle, 13 (28%) in the second week, 13 (28%) in the third week, and 13 (28%) in the fourth week. Among 101 breast cancer patients, 90 had infiltrating ductal carcinoma; three, infiltrating lobular carcinoma; six, ductal carcinoma in situ; one, adenoid cystic carcinoma; and one, sarcomatoid carcinoma. Among 101 patients with benign lesions, 68 had mastopathy; 22, fibroadenoma; eight, intraductal papilloma; and three mastadenitis. Tumor size data for cancer patients was shown in Supplementary Table S1.

**Table 1:** Clinicopathological characteristics of the patients by study group

Variables		Breast Cancer		Benign		Normal	
		Premenopausal (N=47)	Menopausal (N=54)	Premenopausal (N=47)	Menopausal (N=54)	Premenopausal (N=47)	Menopausal (N=54)
**Age, median(range)**		40(24-50)	56(46-78)	40(26-50)	56(47-74)	41(24-53)	56(46-78)
**Family history of breast cancer**		3	4	1	3	1	2
**History of ovarian cancer**		0	0	0	1	0	0
**HT history**		0	0	0	0	0	0
**Prior biopsies**		19	22	16	21	0	0
**menstrual cycle**							
	week1	8	NA	8	NA	8	NA
	Week2	13	NA	13	NA	13	NA
	Week3	13	NA	13	NA	13	NA
	Week4	13	NA	13	NA	13	NA
**Type**							
	luminal A	3	3	NA	NA	NA	NA
	luminal B	31	34	NA	NA	NA	NA
	HER-2 overexpress	12	12	NA	NA	NA	NA
	basal like	1	5	NA	NA	NA	NA

NA, non-applicable; HER-2, human epidermal growth factor receptor 2, HT hormonal therapy

### BPER was associated with menopausal status in a time-dependent manner

**Figure 1:**
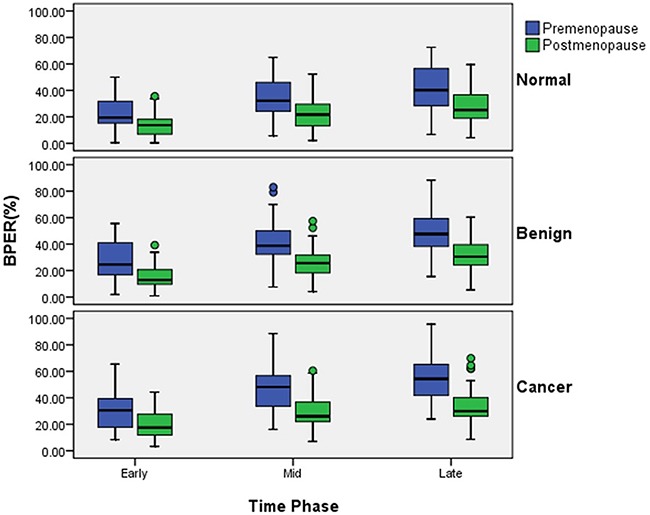
Median BPER of premenopausal and postmenopausal women in the control, benign and cancer groups at the early, medium and late enhanced phases Lines inside the boxes indicate median values, and boxes represent 25th and 75th percentile values. Whiskers show minimum and maximum values.

The median premenopausal BPER was significantly higher than the postmenopausal one (P<0.01), regardless of the phase (early, medium and late enhanced phases) or group (control, benign and breast cancer groups). In both premenopausal and postmenopausal women, BPER increased with increasing enhanced time (Figure 1).

**Figure 2:**
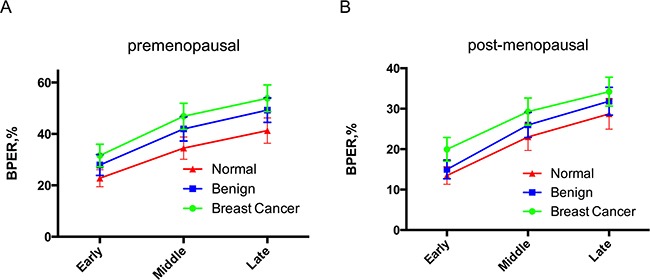
Comparison of premenopausal and postmenopausal BPER in control, benign and breast cancer groups

In premenopausal women, the highest median BPER was observed in the cancer group, meanwhile BPER in the control group was the lowest in the three time phases. The general trend of the three groups was consistent. Additionally, there was an ascending trend with increasing scanning time (Figure 2). The change in BPER of postmenopausal women was similar to that of premenopausal women. In postmenopausal women, BPER was the lowest in the control group, but relatively higher in the cancer group. In the early enhanced phase, the median BPER in the benign group was close to the one in the control group, but at the medium and late phases, it was close to the one in cancer group (Figure 2).

### The cutoff point and OR for breast cancer

The AUC value of BPER in the three phases was compared between cancer and control groups and cancer and benign groups among premenopausal and postmenopausal women. The time phase when the maximum AUC occurred was selected (Table 2). In both premenopausal and postmenopausal women, BPERs in the three time phases were significantly different between cancer and control groups (P<0.05). When the maximum AUC occurred, the time phases corresponded to the medium enhanced phases in premenopausal women and early enhanced phases in postmenopausal women. With maximum AUC, the cutoff point of BPER between the cancer and control groups was 40.3% (sensitivity 65.96%; specificity 68.09%) and 10.05% between the premenopausal and postmenopausal women (sensitivity 87.04%; specificity 40.74%). There was a significant difference of the BPER between the cancer and benign groups at the medium enhanced phase in premenopausal women and the early enhanced phase in postmenopausal women. The cutoff points in these two phases were 51.4% (sensitivity 46.81%; specificity 78.72%) in premenopausal women and 13.3% (sensitivity 72.22%; specificity 46.30%) in postmenopausal women.

**Table 2:** Comparisons of AUC for the discrimination between breast cancer and benign tissue by BPER

	Menopause status	Phase	AUC	95%CI	P	Cutoffpoint	Sensitivity	Specificity
Cancer vs normal	Premenopausal	Early	0.665	0.555-0.775	0.006			
		Mid	0.704	0.599-0.808	0.001	40.3%	65.96%	68.09%
		Late	0.687	0.580-0.794	0.002			
	Postmenopausal	Early	0.668	0.567-0.769	0.003	10.05%	87.04%	40.74%
		Mid	0.647	0.543-0.751	0.008			
		Late	0.631	0.524-0.738	0.019			
Cancer vs benign	Premenopausal	Early	0.554	0.437-0.672	0.364			
		Mid	0.622	0.509-0.736	0.041	51.4%	46.81%	78.72%
		Late	0.581	0.465-0.698	0.173			
	Postmenopausal	Early	0.633	0.529-0.738	0.017	13.3%	72.22%	46.3%
		Mid	0.565	0.456-0.674	0.244			
		Late	0.545	0.436-0.654	0.421			

AUC, area under the curve; BPER, background parenchymal enhancement rate; CI, confidence interval

The breast cancer risk of women with BPER above the cutoff point was 4.1 times higher than for normal premenopausal women, 4.6 times higher than for normal postmenopausal women (Table 3), 2.6 times higher than for premenopausal women with benign lesions and 2.8 times higher than for postmenopausal women with benign lesions (Table 4).

**Table 3:** ORs for the comparison of cutoff points between the cancer and control groups

BPER	Breast Cancer	Normal	OR (95% CI)	P
Premenopausal (N=47)				0.001
<40.3%	16 (34)	32 (68)	1.0	
≥40.3%	31 (66)	15 (32)	4.1 (1.7-9.7)	
Postmenopausal (N=54)				0.001
<10.05%	7 (13)	22 (41)	1.0	
≥10.05%	47 (87)	32 (59)	4.6 (1.7-12.0)	

BPER, background parenchymal enhancement rate; CI, confidence interval; OR, odds ratio

**Table 4:** ORs for the comparison of cutoff points between cancer and benign groups

BPER	Breast cancer	Benign lesions	OR (95% CI)	P
Pr-menopausal (N=47)				0.03
<51.4%	26(55)	36(77)	1.0	
≥51.4%	21(45)	11(23)	2.6 (1.1, 6.4)	
Postmenopausal (N=54)				0.011
<13.3%	16(30)	29(54)	1.0	
≥13.3%	38(70)	25(46)	2.8 (1.2, 6.1)	

BPER, background parenchymal enhancement rate; CI, confidence interval; OR, odds ratio

## DISCUSSION

Previous study reported the relationship between FGT/BPE and BI-RADS grade with visualization assessment [10]. Recently, Wengert GJ compared the quantitative and visualization measurements of FGT and suggested that quantitative measurements can provide reliable and standardized assessment of FGT with MRI [17]. Our study showed FGTs were only significantly different between postmenopausal breast cancer patients and normal population (Supplementary Table S2. Therefore, we designed a quantitative software to assess BPE.

In the present study, we compared BPER in breast cancer, benign lesions and control cohorts of patients who were not receiving hormone replacement therapy or hormonal deprivation therapy. Increased BPER was highly associated with breast cancer after adjusting for age, menopausal status and menstrual cycle. The most significant cutoff point was observed at the middle enhancement time phase for premenopausal women and at the early enhancement time phase for postmenopausal women.

Regions with increased BPE represent breast tissue with high biological activity, where carcinogenesis is thought to occur more frequently [18]. In our study, regardless of the menopausal status, breast cancer patients had higher BPER than those in the benign or control cohorts. These results are consistent with the observations of King [9] and Dontchos [10].

Previous pilot studies suggested that premenopausal women would have higher BPE and that BPE increased as the time of enhanced MRI increased [12–14]. This time-dependent trend of BPER was not only observed in breast cancer, but also in the benign and control cohorts. The same findings were observed in our study.

The most discriminative time phases of BPER for breast cancer prediction were different for pre- and postmenopausal populations. As far as we know, this was the very first study to arrive at this conclusion. In our study, the most diverse BPER value between the breast cancer and benign lesions groups among premenopausal women occurred at the middle time phase of enhanced MRI. The cutoff point was 40.3% for the control group and 51.4% for the benign lesion group. The risk of breast cancer morbidity in women with a BPER higher than the threshold would have been 310% and 160% higher in the future, respectively. These findings differed from those in the postmenopausal women in both time phase and threshold. We consider that the early phase would be better to distinguish breast cancer risk with the highest AUC value. At the middle and late phases, postmenopausal women with benign and malignant lesions had significantly higher BPER than controls. However, there were no significant differences of BPER between the benign and cancer cohorts. The cutoff point was 10.05% for the control group and 13.3% for the benign lesion group. The risk of breast cancer morbidity in women who had a BPER higher than the threshold would have been 360% and 180% higher in the future, respectively. Based on these observations, we recommend that when predicting breast cancer risk using BPER, patients should first be categorized according to their menopausal status.

Gail's model was the most widely used breast cancer risk predictor, with an AUC of 0.602 to 0.670. After adding the parameter of mammary gland density, the prediction accuracy increased to 0.620 to 0.680 [19–23]. Furthermore, combining single-nucleotide polymorphisms (SNPs) factors to this algorithm, the AUC is 0.61 to 0.63 [24]. In the present study, BPER had a higher prediction capability by itself, with an AUC of 0.668 to 0.704.

A major advantage of our study is that BPER is a minimally invasive assessment that allows the early detection of breast cancer. However, there were several limitations to this study. First, enhanced MRI was only divided into three time phases with low-time resolution. Further studies with tensor scanning would improve MRI time resolution with more discriminative cutoff points. Second, the software for BPE applied in this study is still not commercially available and can only be used in specific work stations. Wide application of this model is warranted. Third; BRCA1/2 mutations were associated with breast cancer, however in this retrospective study BRCA 1/2 status was not available. Our study showed that BPER is a predictor of high risk of developing breast cancer. Additionally, the individualized cutoff point of BPER for differentiation breast cancer by menopausal status makes the model more accurate.

## MATERIALS AND METHODS

### Study design and patients

In this study, we retrospectively collected consecutive 14,033 women underwent breast MRI examination in our hospital from 2009 to 2012. Among them, only 391 individuals were bilateral normal breast (BI-RADS 1) in MR test and without any lesion on MRI, or mammography, or ultrasound examination 2 times in subsequent two years. Only patients with 4 weeks menstrual cycle without hormonal therapy were enrolled. There were 101 control subjects meeting the criteria. Based on this selected control group, we selected age- and menstrual status-matched with 4 weeks of menstrual cycle patients with breast cancer and benign lesions with a ratio of 1:1. The age ranges were defined as less or equal to 5 years. Patients were categorized based on premenopausal and postmenopausal status. Premenopausal status was subclassified into four groups (i.e., 1–7 days after menstruation for the 1st week, 8 to 14 days for the 2nd week, 15 to 21 days for the 3rd week, and 22 days to the next menstruation time for the 4th week) based on the menstrual cycle. Patients with breast cancer had untreated, unilateral breast cancer, whereas the patients with benign lesions had unilateral breast benign lesions confirmed by biopsy or operation.

### MRI data collection

All breast MRI examinations were performed with an Aurora 1.5-T dedicated breast MRI scanner (Aurora Imaging Technology, Inc., Canada) and Breast unique transmit/receive coil (two channels). Patients were in prone position, and both breasts were examined with natural ptosis. The scanning range included bilateral breast and axillary areas. The contrast used to acquire the dynamic contrast enhancement MRI (DCE-MRI) was Gd-DTPA at a dose of 0.2 mmol/kg. The contrast was injected intravenously with a high pressure syringe at a flow rate of 2.0 mL/s. Subsequently, 15 mL of 0.9% NaCl solution was injected at the same flow rate to flush the remaining Gd-DTPA. Each case underwent DCE-MRI, which typically included several MRI series, including one-position reference image, one T2-weighted fat-suppressed image, one T1-weighted nonfat-suppressed image, one pre-contrast T1-weighted fat-suppressed image and three post-contrast T1-weighted fat-suppressed images. The post-contrast images were acquired at three time phases (early-2 minutes, middle-4 minutes, and late phases-6 minutes) after contrast injection. The scanning time for the pre-contrast or each post-contrast MRI series was about 2 minutes. The scanning parameters are shown in Table 5.

**Table 5:** Scanning parameters of 1.5T dedicated breast MRI systems

MRI Sequence	TE (msec)	TR (msec)	Field of View (cm)	Section Thickness (mm)	Matrix
Scout	8.8	20.0	36	160	192×64
Axial T2-weighted fat suppressed	68	4008	36	5	320×256
Axial T1-weighted non–fat suppressed	5.3	12.9	36	5	285×256
Axial T1-weighted fat suppressed	4.8	29.0	36	1.5	360×360

Abbreviations: MRI, magnetic resonance imaging; TE, echo time; TR, repetition time

### MRI data evaluation

**Figure 3:**
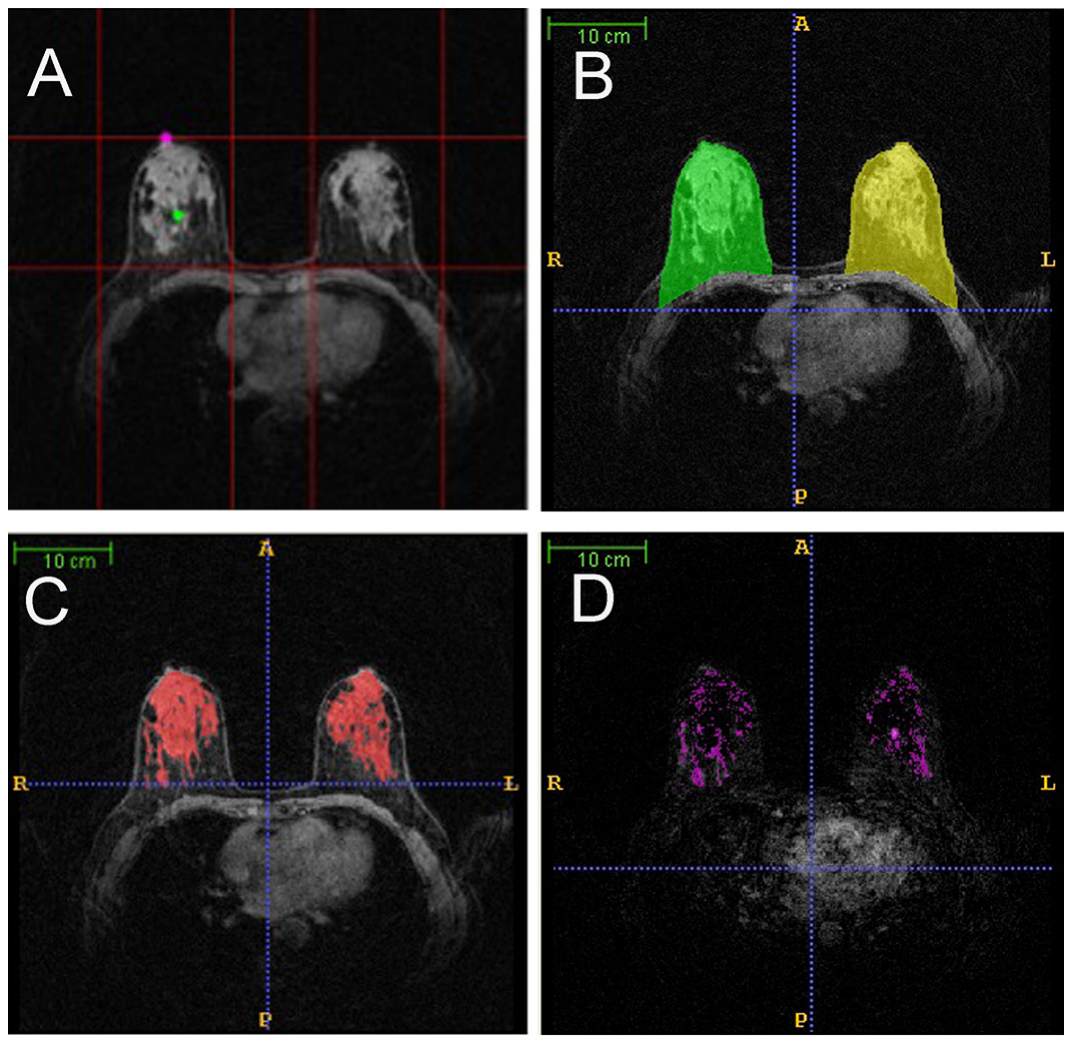
Magnetic resonance imaging analysis **A**. volume of interest (VOI) of breasts; **B**. whole breast segmentation (green for the right breast and yellow for the left breast); **C**. fibroglandular tissue segmentation (red); **D**. enhanced fibroglandular tissue segmentation (pink).

We developed a fully automated scheme for the quantitative analysis of BPE in images obtained by DCE-MRI. Our fully automated method consists of three steps, that is, segmentation of the whole breast, fibroglandular tissues, and enhanced fibroglandular tissues. Based on the volume of interest extracted automatically, a dynamic programming method was applied in each two-dimensional slice of a 3D MRI scan to delineate the chest wall and breast skin line for segmenting the whole breast. This step took advantage of the continuity of the chest wall and breast skin line across adjacent slices. We then further used the fuzzy c-means clustering method with an automatic selection of cluster numbers for segmenting the fibroglandular tissues within the segmented whole breast area. Finally, a statistical method was used to set a threshold based on the estimated noise level for segmenting the enhanced fibroglandular tissues in the subtraction image of pre- and post-contrast MRI scans (shown in Figure 3).

Background parenchymal enhancement rate (BPER) was the volume ratio of the enhanced fibroglandular tissues and the fibroglandular tissues.

BPER=Ve/Vt×100%

Ve: enhanced fibroglandular tissue volume

Vt: total fibroglanduar tissue volume

### Statistical analysis

According to the relative consistency of background parenchyma in bilateral breasts, to avoid the effects of the lesion itself, the mean between bilateral breasts was chosen as the measurement target in the control group, as well as contralateral breast in benign and cancer groups. The BPER in premenopausal and postmenopausal women was compared using the Mann–Whitney U test. Receiver operating characteristic (ROC) curves of BPER in premenopausal and postmenopausal women were drawn respectively between cancer and control groups and cancer and benign groups using a nonparametric method. The AUCs in the three phases after enhancement were calculated to find the optimal phase of breast cancer-related BPER. With the application of the ROC curve of the optimal phase and the cutoff point were chosen with the maximum AUC. The ORs between the cancer and control groups and the cancer and benign groups were estimated.

## SUPPLEMENTARY TABLES


