# Supplementary material for: Quantitative assessment of background parenchymal enhancement in breast magnetic resonance images predicts the risk of breast cancer
Source: Oncotarget. 2016 Nov 24;8(6):10620–7. doi: 10.18632/oncotarget.13538 (PMC5354686; doi:10.18632/oncotarget.13538)
Supplement: Supplementary file 1 [file oncotarget-08-10620-s001.pdf]

Quantitative assessment of background parenchymal enhancement in breast magnetic resonance images predicts the risk of breast cancer

SUPPLEMENTARY TABLES

Supplementary Table S1: Tumor size data for cancer patients

Tumor size	Premenopausal(N=47)	menopausal(N=54)
<2cm	10	15
≥2cm, ≤5cm	32	39
>5cm	5	0

Supplementary Table S2: Comparisons of AUC for the discrimination between breast cancer and benign tissue by FGT

Menopause status	Cancer vs Normal				Cancer vs Benign			
	Cancer (%)	Normal (%)	P	AUC	Cancer (%)	Benign (%)	P	AUC
Premenopausal	16.4	17.3	0.689	0.524	16.4	17.0	0.768	0.518
Postmenopausal	9.4	8.0	***0.035***	***0.618***	9.4	10.7	0.749	0.482

FGT, fibroglandular tissue
